# A new stock market analysis method based on evidential reasoning and hierarchical belief rule base to support investment decision making

**DOI:** 10.3389/fpsyg.2023.1123578

**Published:** 2023-02-09

**Authors:** Yujia Chen, Jiangdan Liu, Yanzi Gao, Wei He, Hongyu Li, Guangling Zhang, Hongwei Wei

**Affiliations:** ^1^School of Computer Science and Information Engineering, Harbin Normal University, Harbin, China; ^2^School of Economics, Beijing International Studies University, Beijing, China; ^3^Institute of Advanced Materials and Technology, University of Science and Technology Beijing, Beijing, China

**Keywords:** stock market analysis, evidential reasoning, belief rule base, stock market evaluation, decision making

## Abstract

Stock market analysis is helpful for investors to make reasonable decisions and maintain market stability, and it usually involves not only quantitative data but also qualitative information, so the analysis method needs to have the ability to deal with both types of information comprehensively. In addition, due to the inherent risk of stock investment, it is necessary to ensure that the analysis results can be traced and interpreted. To solve the above problems, a stock market analysis method based on evidential reasoning (ER) and hierarchical belief rule base (HBRB) is proposed in this paper. First, an evaluation model is constructed based on expert knowledge and ER to evaluate stock market sentiment. Then, a stock market decision model based on HBRB is constructed to support investment decision making, such as buying and selling stocks and holding positions. Finally, the Shanghai Stock Index from 2010 to 2019 is used as an example to verify the applicability and effectiveness of the proposed stock market analysis method for investment decision support. Experimental research demonstrates that the proposed method can help analyze the stock market comprehensively and support investors to make investment decisions effectively.

## 1. Introduction

The stock market is an important place for investment and trading. Its structure and trading activities are complex and influential. It is the thermometer of national economic development (Jin and Guo, 2021). The factors that affect the volatility of the stock market are very complex, showing highly nonlinear and dynamic characteristics (Barra et al., 2020), which increases the difficulty and risk of investment. Therefore, for investors to obtain greater returns and avoid investment risks, it is necessary to conduct a reasonable analysis of the stock market (de Oliveira et al., 2011; Chen et al., 2018; Feng et al., 2019).

Many scholars have researched the analysis of the stock market. Broadly speaking, there are two typical approaches of studying the stock market in literature: the seminal and widely studied approach is to make use of statistical analysis, financial theories and knowledge for stock market analysis, while the latest advance in the fields is to explore the use of machine learning and Artificial Intelligence (AI) techniques. Most of the statistical analysis is based on quantitative data. For example, Arashim (2022) proposed an ARMA-GARCH model to analyze the daily stock index of the Nasdaq Stock Exchange. Al-Ani and Zubaidi (2021) used the Kolmogorov–Smirnov test, normality test and Heteroskedasticity test to verify the expected risk of the Iraqi stock market. He et al. (2020) used OLS regression and quantile regression to study the relationship between investor sentiment and stock market volatility. Salman and Ali (2021) studied the impact of COVID-19 on the stock market through conventional t tests and nonparametric Mann–Whitney tests.

With the development of machine learning and AI techniques, scholars have applied relevant algorithms to stock market prediction, such as neural networks, support vector machines (SVM), and Bayesian models. The research on analyzing stocks based on machine learning can be further divided into two categories: regression and classification. Regression analysis focuses on predicting future stock prices by training a large amount of historical data. From the point of view of forecasting stock prices, Zheng and He (2021) proposed a hybrid forecasting model based on principal component analysis (PCA) and a recurrent neural network (RNN) to predict airline stock prices. Chen et al. (2021) constructed a machine learning hybrid model and selected 19 technical indicators to quantitatively predict stock prices. The above algorithms have advantages in solving the high-dimensional feature problem, which is also consistent with the multi-feature data in the field of stock prediction. However, when this method is applied to large-scale training samples, it will consume a lot of computer memory and computing time. Cheng and Chen (2018) proposed a fuzzy time series forecasting model based on weighted association rules to predict the price of gold in financial markets. Classification studies focus on predicting stock trends, which will be divided into two categories: up or down. Common classification algorithms are KNN, support vector machines (SVM), and random forests (RF). From the point of view of trend prediction, Yuan et al. (2020) proposed using feature selection and a machine learning integrated algorithm to predict the trend of China’s stock market. After comparison with many methods, the RF model shows some advantages in predicting the long-term stock price trend. Malagrino et al. (2018) took the closing direction of an index as the changing trend of the closing price of a certain trading day compared with the closing price of the previous day and predicted such a changing trend in combination with a Bayesian network. Ananthi and Vijayakumar (2021) judge the selling or buying trading behavior after predicting the stock market trend through candlestick pattern detection and the KNN algorithm. Xiao et al. (2020) combined SVM with the traditional ARIMA model to predict the direction of stock price fluctuations. Although algorithms such as SVM have been successfully used to predict financial time series, there are some limitations to these methods. For example, stock market data is characterized by huge noise, non-smoothness and complex dimensionality (Kara et al., 2011). The algorithm often shows unpredictable performance on noisy data. It imposes a significant challenge in predicting stock trends. For investors, the ultimate purpose of analyzing the stock market is to make investment decisions. In fact, this is a problem of classification. In practice, this is a matter of classification. Therefore, it is necessary to study an efficient classification algorithm to analyze stocks.

Through the above analysis of literature, the following challenges and limitations should be considered rigorously in the process of stock analysis. First, in the process of making investment decisions, investors need to analyze crossings and fluctuations between indicator lines. For example, a gold cross is regarded as an important signal to buy a stock, while most of the above research performances analysis from a quantitative perspective and ignores the piece of qualitative information. Second, the stock market is complex and nonlinear, so the model performance should be measured appropriately in the process of analysis (He et al., 2020). Finally, most stock analysis methods based on machine learning are data-driven models. The analysis process is not interpretable, and the results are not traceable.

Based on the above analysis, it is of great significance to construct an effective market analysis model to cover both stages of evaluating the stock market and making investment decisions such as buying and selling stocks. During the stock market evaluation process, investors must consider many factors, including both quantitative and qualitative information. In order to comprehensively analyze the above information, this paper adopts a evaluation method based on evidential reasoning (ER) proposed by Yang et al. (2006). The ER is a multi-attribute decision analysis method based on evidence (Kong et al., 2015). The stock market can be reasonably assessed. In the comprehensive decision-making of the stock market, the idea of the belief rule base (BRB) is adopted. This method was proposed by Yang and Xu (2013) BRB is based on IF-THEN rule. Expert knowledge can be used to constrain these rules and set the initial parameters of the model. As a result, the relationship between the inputs and outputs is explained. BRB itself can be seen as an expert system that can effectively deal with problems such as fuzzy uncertainty and probabilistic uncertainty. The BRB model can establish a complex nonlinear relationship between input and output according to the financial mechanism (Zhou et al., 2016). Different from machine learning algorithms such as neural networks and SVM, the reasoning process of BRB can be verified and explained (Hossain et al., 2018). ER and BRB are homologous methods. Therefore, the original physical meaning will not be destroyed, and the information will not be lost in use. The modeling method based on ER and BRB is also applied to medical analysis (Kong et al., 2012), fault diagnosis (Cheng et al., 2022), natural gas pipeline leak assessment (Feng et al., 2021) and so on.

In this paper, a method based on the ER algorithm and hierarchical BRB is proposed to analyze the stock market, and the main contributions can be summarized as follows:

1. In this paper, an interpretable stock analysis model based on ER and hierarchical BRB is proposed. The reasoning process of the model is causal, and the results can be traced back (Cao et al., 2021). In stock analysis, the process is transparent and the results are more convincing.
2. The proposed stock market evaluation model can deal with both quantitative and qualitative information. It can comprehensively consider the subjective uncertainty of qualitative indicators and the probabilistic uncertainty of quantitative indicators.
3. To comprehensively analyze the stock market, a hierarchical BRB model based on the weight of attributes is proposed in this paper. The accuracy of the model can be improved through top-level training of data. Besides, the model is extensible, and the problem of rule explosion in multiple attributes is reduced.

The structure of the paper is as follows. In Section 2, the key tasks in stock market analysis are formulated. In Section 3, a stock market analysis model is constructed. In Section 4, the validity of the constructed model is verified. A summary of the full text and future work are given in Section 5.

## 2. Problem formulation

The constructed stock market analysis model mainly comprehensively analyzes multiple types of technical indicators to provide investors with investment decisions, such as whether they should sell stocks, buy stocks or hold positions in the current situation. Feature extraction and evaluation of the current stock market quotation are also required before comprehensive decision making. The key tasks are described below.

### Task 1: Feature extraction of stock market data

In the process of market analysis, the data of the stock market cannot reflect the situation of the stock market, and it is necessary to mine the internal characteristics of different indicators. Therefore, selecting a set of appropriate features from a large number of data $x_{m}\cdots x_{n}$ is the most important problem to be solved. The process of feature extraction is described as follows.


(1) $$a_{i}\left(t\right)=S\left(x_{m}\left(t\right)\cdots x_{n}\left(t\right),\lambda \right)$$

where $a_{i}\left(t\right)$ denotes the ith feature extracted at moment t, S(•) denotes the feature extraction method, and λ is the time period. There are many types of common stock technical indicators, such as moving averages (MA), moving average convergence divergence (MACD), relative strength index (RSI), on balance volume (OBV), etc. In this paper, three types of indicators, MA, MACD and stochastic Indicator (KDJ), are selected for stock analysis.

### Task 2: Processing of quantitative and qualitative information

Before making an investment decision, a primary analysis of stock market quotation based on different types of indicator data is performed. Market quotation is quantified in preparation for making investment recommendations. The stock market has the characteristics of uncertainty and nonlinearity. At the same time, both quantitative information and qualitative information are included in the analysis process. How to construct a model that can deal with both qualitative and quantitative data and nonlinearly reflect the results of stock market evaluation is a problem that needs to be solved, which is described as follows:


(2) $$z_{j}\left(t\right)=ER\left(a_{i}\left(t\right),\chi \right)\;1\leq z_{j}\left(t\right)\leq 5$$

where $z_{j}\left(t\right)$ is the output of the jth stock market evaluation model at time t and the value is between 1 and 5. The larger the value is, the more depressed the market is, and the stock should be sold at this time. ER(•) denotes the nonlinear conversion process from stock market index data to stock market evaluation results. χ denotes the parameters set according to the actual situation of the stock market. The parameters set contains the weights of the attributes. Based on the extracted features, three ER models are constructed in this paper, and the stock market evaluation results of each model are $z_{1}\left(t\right)$, $z_{2}\left(t\right)$, $z_{3}\left(t\right)$.

### Task 3: Comprehensive multi-index stock market analysis

When investors actually analyze the stock market, they often need to consider many factors comprehensively. In addition, a large amount of financial knowledge and investment experience needs to be incorporated to obtain reasonable and reliable investment recommendations. Therefore, the problems of how the model can combine expert knowledge to achieve multi-attribute decision making and provide investors with reasonable recommendations are to be addressed. The process is described as follows.


(3) $$y\left(t\right)=BRB\left(z_{j}\left(t\right),\eta \right)$$

where y(t) means to provide investors with trading recommendations on date t, and the trading recommendations include five aspects: buy all positions, buy half positions, hold a position, sell half positions, and sell all positions. BRB(•) denotes the nonlinear transformation process from the results of single-type technical index analysis to the results of comprehensive analysis. η denotes the parameters set of the model, which includes the weight of attributes and the weight of rules.

## 3. Construction of stock analysis model

To solve the above tasks, a stock market analysis model is constructed in this section. Stock analysis model mainly includes stock market evaluation model and stock market comprehensive decision-making model. The overall structure of the model is described in Section 3.1. The technical indicators selected for this paper are presented in Section 3.2. In Section 3.3, the process of implementing the stock market analysis model is described in detail.

### 3.1. The overall structure of the model

**Figure 1:**
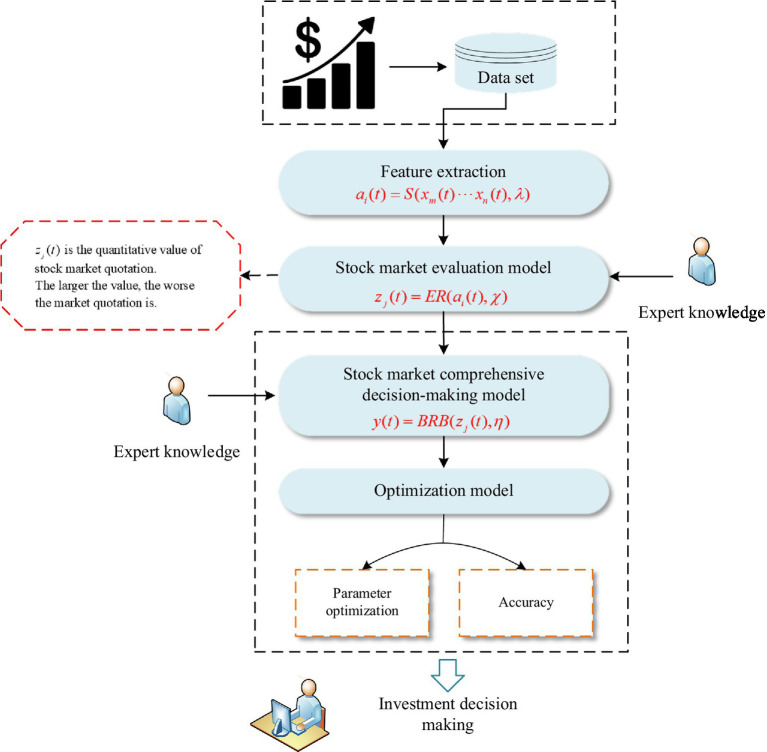
The structure of the stock analysis model.

The overall structure mainly includes four parts: feature extraction, stock market evaluation model, stock market comprehensive decision-making model and optimization model. The structure of the stock analysis is shown in Figure 1.

First, the model analyzes the relationship between various technical indicators according to the stock mechanism for feature extraction. Second, based on the features extracted above, a stock market evaluation model is constructed. This model is used to assess whether the stock market sentiment is optimistic. Next, when the stock market evaluation is completed, a stock market comprehensive decision-making model is constructed. The model takes the evaluation results as input, and the model can output investment decisions. Among them, investment decisions include buying stocks, holding stocks and selling stocks. Finally, to improve the accuracy of the analysis results, an optimization model is constructed, and the model parameters are continuously optimized.

### 3.2. Feature extraction

The most directly and easily available technical indicators in stock data include opening price, closing price, highest price and lowest price. To analyze the stock market comprehensively from different perspectives, a large number of technical indicators have been introduced on this basis. From a functional perspective, technical indicators can be generally divided into the following three categories: trend indicators, overbought and oversold indicators, and energy indicators.

The trend indicator is used to judge the changing trend of stock prices. It can eliminate the effect of short-term changes and other accidental factors on the change of stock price. The common trend indicators are the moving average (MA) (Tanaka-Yamawaki and Tokuoka, 2007), moving average convergence divergence (MACD) (Wang and Kim, 2018) and so on. The overbought and oversold trading indicator is not only an indicator to judge the trend of market price and the phenomenon of overbought and oversold but also a technical indicator for short-term investment. The common overbought and oversold indicators are the Williams percent range and stochastic oscillator (KDJ). Energy indicators refer to the observation of stock price changes mainly from the perspective of volume. For example, on balance volume (OBV) and volume ratio (VR) are all energy indicators.

**Figure 2:**
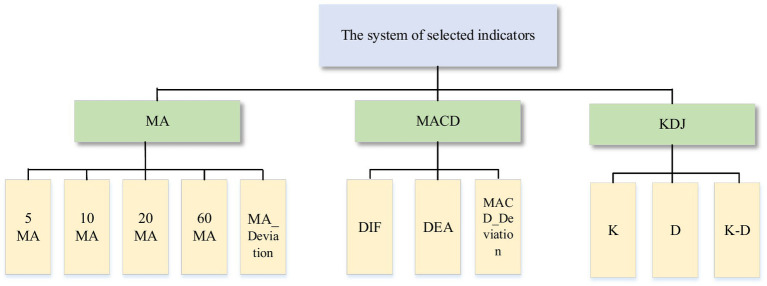
The system of selected indicators.

In this paper, MA, MACD and KDJ are selected to analyze the stock market. The reasons are as follows: first, MA has the characteristics of trend and smoothness, which is an important reference to judge the trend of stock prices. Second, MACD not only has the advantage of MA but also reacts more significantly to recent price changes (Aguirre et al., 2020). Third, MA and MACD have lags (Anghel, 2015; Wu and Diao, 2015), so this paper also analyzes the KDJ. This type of indicator can quickly and intuitively analyze the market and make judgments on buying and selling. Figure 2 shows the system of selected indicators. The following is the calculation method of the indicator.

#### 3.2.1. Moving average

A moving average is the arithmetic average of the closing prices of a number of consecutive days, which is used as a tool of price trend. Another measure is deviation, which is the degree of deviation between the closing price and a particular moving average. The formulas are as follows:


(4) $$nMA=\frac{\sum_{i=1}^{n}close_{i}}{n}$$

(5) $$nMA\_\text{Deviation}=\frac{{\text{close}}_{i}-nMA}{nMA}\times 100\%$$

where n represents the period of the indicator. ${\text{close}}_{i}$ represents the closing price on the ith day, and nMA_Deviation represents the MA deviation rate on n days.

#### 3.2.2. Moving average convergence divergence

MACD is a technical indicator that reflects the aggregation and separation between the 12-day exponential moving average (EMA) and the 26-day exponential moving average. It is an important indicator to judge when to buy and sell. Differential value (DIF) is the difference between the 12-day EMA and the 26-day EMA. Difference exponential average (DEA) is the average DIF over 9 days. The formulas are as follows:


(6) $$12\_EMA=\left(\text{close}\times 2+12\_EMA^{’}\times \left(12-1\right)\right)/\left(12+1\right)$$

(7) $$26\_EMA=\left(\text{close}\times 2+26\_EMA^{’}\times \left(26-1\right)\right)/\left(26+1\right)$$

(8) DIF=12_EMA−26_EMA

(9) $$DEA=\left(DEA\times 2+DEA^{’}\times \left(9-1\right)\right)/\left(9+1\right)$$

(10) MACD=(DIF−DEA)×2

(11) $$MACD\_\text{Deviation}=\frac{MACD-\sum_{i=1}^{60}MACD_{i}/60}{\sum_{i=1}^{60}MACD_{i}/60}$$

where close represents the closing price of the day.12_EMA and 26_EMA represent the 12-day exponential moving average and the 26-day exponential moving average, respectively. $12\_EMA^{’}$ and $26\_EMA^{’}$ represent exponential moving average of the previous days. MACD_Deviation indicates the deviation of the MACD from the 60-day moving average on that day, where $MACD_{i}$ denotes the MACD value of the previous i days.

#### 3.2.3. Stochastic indicator

When calculating the KDJ indicator, the row stochastic value (RSV) of the day is calculated first, and then the K and D values are calculated according to this value. The calculation formulas are as follows:


(12) $$RSV_{i}=\frac{\text{Close}-{\text{Low}}_{i}}{{\text{High}}_{i}-{\text{Low}}_{i}}\times 100$$

(13) $$K_{i}=\frac{2}{3}K_{i-1}+\frac{1}{3}RSV_{i}$$

(14) $$D_{i}=\frac{2}{3}D_{i-1}+\frac{1}{3}K_{i}$$

where close represents the closing price of the day. ${\text{Low}}_{i}$ and ${\text{High}}_{i}$ represent the lowest and highest prices on the ith day, respectively. $K_{i}$ and $D_{i}$ are the K and D values of the ith day, respectively.

### 3.3. Implementation of the model

**Figure 3:**
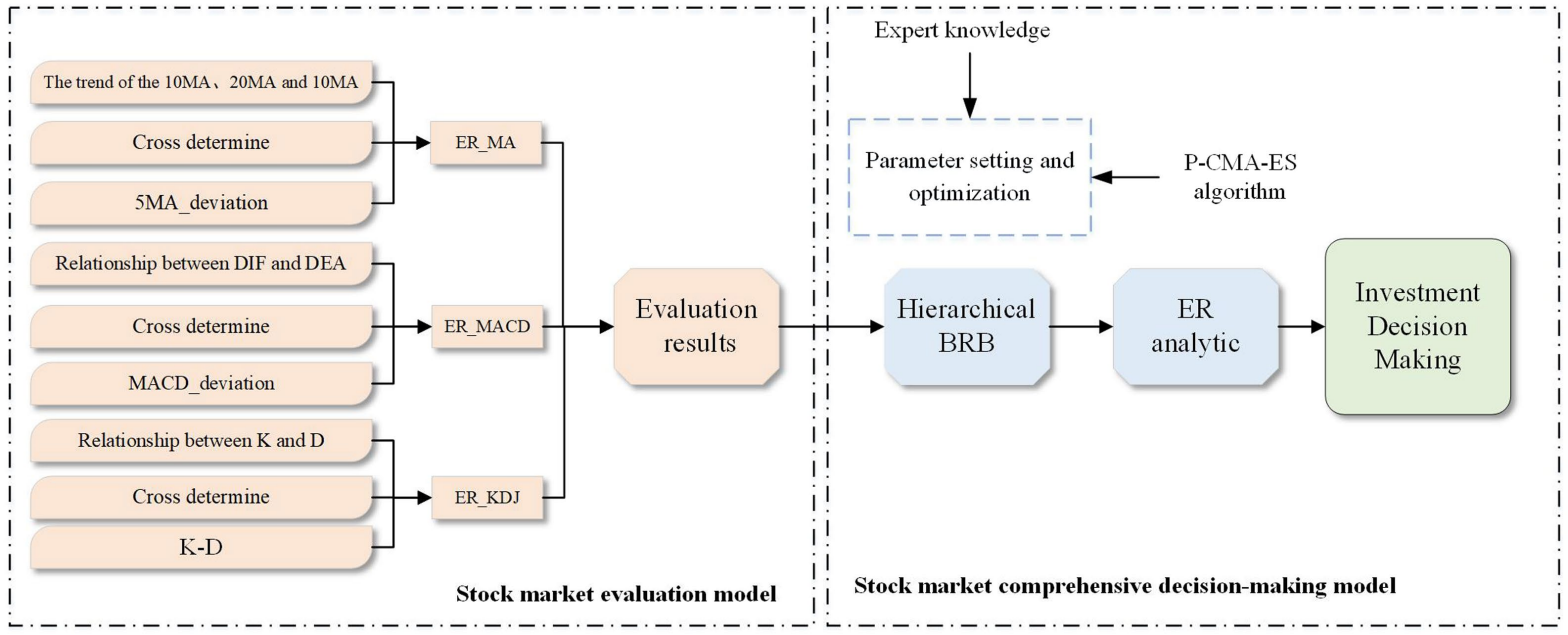
The implementation process of the stock analysis model.

A stock market evaluation model based on ER and a stock market comprehensive decision-making model based on hierarchical BRB are constructed. According to the selected features, the stock market evaluation model is further divided into three parts: the ER_MA model, ER_MACD model and ER_KDJ model. The implementation process of the stock analysis model is shown in Figure 3.

#### 3.3.1. Stock market evaluation model

This section mainly refers to the indicators MA (5 MA, 10 MA, 20 MA, and 60 MA), MACD (DIF, DEA) and KDJ (K, D) to evaluate the situation of the stock market. The following is the structure of the model and the reasoning process of the model.

##### 3.3.1.1. The structure of the model

Based on the ER iterative algorithm, three models, ER_MA, ER_MACD, and ER_KDJ, are constructed. The model outputs an assessment of the stock market sentiment. The results of this assessment contain three categories: positive (P), intermediate (M) and negative (N). The following are definitions of model attributes.

###### 3.3.1.1.1. ER_MA model

The technical indicator of the MA type is an indicator that describes the trend state of stock prices. In the process of stock trading evaluation, 10MA, 20MA and 60MA are arranged from top to bottom, that is, 10MA > 20MA > 60MA, which reflects the trend of positive development of the stock market. It also means that this is the time to buy the stock. If it is arranged in the opposite direction, it is a negative signal, indicating the timing of selling. Similarly, the golden cross and dead cross are the key points to judge buying and selling. In the ER_MA model, the golden cross and the dead cross need to be based on two indicators: 5 MA and 10 MA. When 5MA crosses 10MA from bottom to top, the intersection is the golden cross. When there is a golden cross, the stock market will have some room to rise, which is a positive signal and the best time to buy. In contrast, it is a dead cross. Attributes can be divided into three levels, namely, negative signal, intermediate signal and positive signal. The attributes are shown in Table 1.

**Table 1:** ER_MA model attributes.

Attribute	Decision rules	The reference level
The trend of the 10MA, 20MA and 10MA	10MA < 20MA < 60MA	N
	Other situations	M
	10MA > 20MA > 60MA	P
Cross determine	Dead cross	N
	Other situations	M
	Golden cross	P
5MA_deviation	5-day deviation: −2%	N
	5-day deviation: −0%	M
	5-day deviation: 2%	P

###### 3.3.1.1.2. ER_MACD model

The technical indicator of MACD type is a famous trend indicator. The DIF and DEA indicators are referenced in the model. When DIF > 0 and DEA > 0, a positive trend is shown in the stock market, which is the buy signal. When DIF < 0 and DEA < 0, a negative trend appears in the stock market, which is a sell signal. In the MACD model, when DIF breaks through DEA from the bottom up, the golden cross is formed, which is the buy signal. When DIF breaks through DEA from top to bottom, a dead cross is formed, which is a sell signal. The attributes are shown in Table 2.

**Table 2:** ER_MACD model attributes.

Attribute	Decision rules	The reference level
Relationship between DIF and DEA	DIF < 0, DEA < 0	N
	Other situations	M
	DIF > 0, DEA > 0	P
Cross determine	Death cross	N
	Other situations	M
	Golden cross	P
MACD_deviation	MACD deviation: −5%	N
	MACD deviation: 0%	M
	MACD deviation: 5%	P

###### 3.3.1.1.3. ER_KDJ model

The technical indicator of KDJ type is an indicator that describes the signals of stock buying and selling. The values of K and D are usually between 0 and 100. When the values of K and D are greater than 80, the market is overbought. When the values of K and D are less than 20, the market is oversold. The K value is greater than the D value; that is, when the K line breaks through the D line upwards, the golden cross point is formed, which is the buy signal. The K value is less than the D value; that is, when the K line falls below the D line, the dead cross point is formed, which is a sell signal. Attributes can be divided into three levels, namely, negative signal, intermediate signal and positive signal. The attributes are shown in Table 3.

**Table 3:** ER_KDJ model attributes.

Attribute	Decision rules	The reference level
Relationship between K and D	0 < K < 20,0 < D < 20	N
	Other situations	M
	80 < K < 100,80 < D < 100	P
Cross determine	Dead cross	N
	Other situations	M
	Golden cross	P
K-D	K-D: −13	N
	K-D: 0	M
	K-D: +13	P

##### 3.3.1.2. Reasoning process

Combined with expert knowledge, the fusion process of multiple indicators is as follows:

Step 1: Initialize the confidence of the evaluation level of financial indicators.

There are M indicators under the model, in which $h_{1},h_{2},\cdots h_{n}$ represents all the levels at which the indicators can be evaluated, namely, $\Theta =\left\{h_{1},h_{2},\cdots h_{n}\right\}$. Then, $\sigma_{r,m}\left(r=1,2,---,R;m=1,2,---,M\right)$ represents the degree of belief that the kth technical indicator is rated as $h_{r}$, which is described as:


(15) $$e_{m}=\left\{\left(h_{r},\;\sigma_{r,m}\right),\left(\Theta_{r},\;\sigma_{\Theta ,m}\right),\;r=1,\;2,\;\cdots \;,\;R;\;m=1,\;2,\;\cdots \;,\;M\right\}$$

$$\text{Remark 1: For example, in the ER\_MACD model, if DIF }<0\text{and DEA }<0,\text{ then}e_{1}=\left\{\left(N,1\right),\left(M,0\right),\left(P,0\right)\right\}.$$

Step 2: Integrate the information of various financial indicators.

The ER is established based on the Dempster rule, which can be used for data fusion (Yang and Xu, 2013). The ER algorithm can assign residual support to any single propositions and the frame of discernment, while Dempster’s combination rule allocates all residual support to the frame of discernment. ER provides an efficient way of expressing information, where uncertainty can be transformed into basic probability mass by certain rules (Zhou et al., 2021). The information is expressed in the form of a belief distribution and can be transmitted without loss in the process of evidence combination. The following is the process of evidence combination.

First, the belief degrees $\sigma_{r,m}$ needs to be transformed into a basic probability mass (Feng et al., 2019). The formula is as follows:


(16) $${\text{mass}}_{r,m}=\omega_{m}\sigma_{r,m}$$

(17) $${\text{mass}}_{h,m}=1-\omega_{m}\sum_{n=1}^{R}\sigma_{r,m}$$

(18) $$m\bar{a}ss_{h,m}=1-\omega_{m}$$

(19) $$m\tilde{a}ss_{h,m}=\omega_{m}\left(1-\sum_{n=1}^{R}\sigma_{r,m}\right)$$

where ${\text{mass}}_{r,m}$ represents the basic probability mass of the mth level of the rth indicator, and $\omega_{m}$ represents the weight of the mth indicator. ${\text{mass}}_{h,m}$ is denoted as the basic probability mass not assigned to the rank set, and ${\text{mass}}_{h,m}=m\bar{a}ss_{h,m}+m\tilde{a}ss_{h,m}$. $m\bar{a}ss_{h,m}$ denotes the basic probability mass of the missing mth indicator. In cases where the expert is unable to give precise rules or where some of the input data is not available, the results of the rules will be incomplete. Therefore, the incompleteness should be taken into account in the reasoning process. $m\overset{\;}{\tilde{a}}ss_{h,m}$ denotes the degree of incompleteness of the mth indicator.

Then, the m rules are combined by using ER and described as follows.


(20) $$\begin{array}{l} {\text{mass}}_{r,e\left(m+1\right)}=\\ Q_{e\left(m+1\right)}\left[\begin{array}{l} {\text{mass}}_{r,e\left(m\right)}{\text{mass}}_{r,m+1}+{\text{mass}}_{r,e\left(m\right)}\\ {\text{mass}}_{h,k+1}+{\text{mass}}_{h,e\left(m\right)}{\text{mass}}_{r,m+1} \end{array}\right] \end{array}$$

(21) $${\text{mass}}_{h,e\left(m\right)}=m\bar{a}ss_{h,e\left(m\right)}+m\overset{\;}{\tilde{a}}ss_{u,e\left(m\right)}$$

(22) $$\begin{array}{l} {\text{mass}}_{h,e\left(m+1\right)}=Q_{e\left(m+1\right)}\\ \left[\begin{array}{l} m\tilde{a}ss_{h,e\left(m\right)}m\tilde{a}ss_{h,m+1}+m\tilde{a}ss_{h,e\left(m\right)}\\ m\bar{a}ss_{h,m+1}+m\bar{a}ss_{h,e\left(m\right)}m\tilde{a}ss_{h,m+1} \end{array}\right] \end{array}$$

(23) $$m\bar{a}ss_{h,e\left(m+1\right)}=Q_{e\left(m+1\right)}\left[m\bar{a}ss_{h,e\left(m\right)}m\overset{\;}{\tilde{a}}ss_{h,m+1}\right]$$

(24) $$Q_{e\left(m+1\right)}=\frac{1}{1-\sum_{r=1}^{R}\sum_{\begin{array}{l} t=1\\ t\neq r \end{array}}^{R}{\text{mass}}_{r,e\left(m\right)}{\text{mass}}_{t,m+1}}$$

where ${\text{mass}}_{r,e\left(m\right)}$ and ${\text{mass}}_{h,e\left(m\right)}$ respectively denote the basic probability mass assigned to the rth evaluation level and the basic probability mass assigned to the identification framework after the combination of m rules.

Finally, the combined belief degree $\sigma_{r}$ is calculated. It can be described as follows:


(25) $$\sigma_{r}=\frac{{\text{mass}}_{r,e\left(m\right)}}{1-m\bar{a}ss_{h,e\left(m\right)}}$$

Step 3: Utility Computing

The evaluation results of the ith model were quantified using the following utility formula.


(26) $$z_{j}\left(t\right)=\sum_{r=1}^{R}u\left(h_{r}\right)\sigma_{r}$$

where u(•) denotes the set of utilities.

Remark 2: ER algorithm has the ability to deal with qualitative and quantitative information comprehensively. The evaluation results are based on formulaic reasoning and are traceable.

#### 3.3.2. Stock market comprehensive decision-making model

The three types of stock indicators MA, MACD and KDJ obtained under the ER algorithm can quantify the status of the stock market, but the evaluation results are inferred by a type indicator. The volatility of the stock market is often influenced by the interaction of many factors. A comprehensive decision-making model for the stock market needs to be constructed. The utility results of the MA model, the MACD model and the KDJ model are used as the premise attributes to construct a hierarchical BRB model.

##### 3.3.2.1. The structure of the model

**Figure 4:**
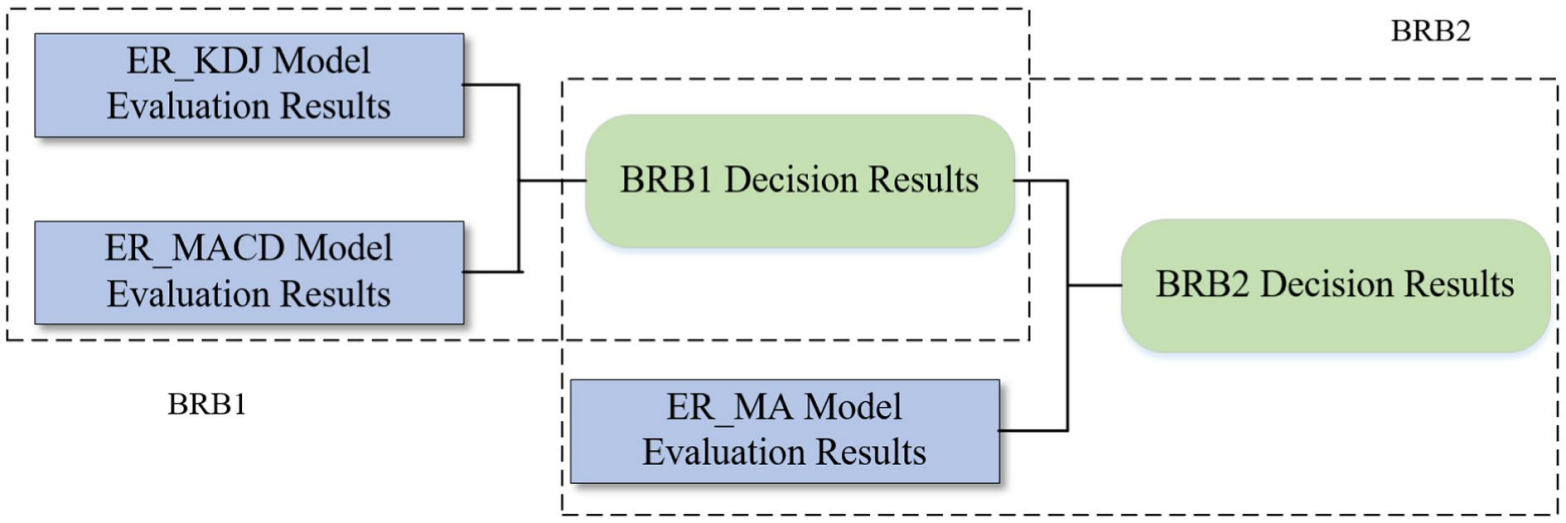
The structure of the hierarchical BRB model.

To solve the rule explosion problem when multiple attributes are input, a hierarchical BRB model can be considered. The model is a bottom-up structure and is easily extensible. The main idea is to arrange the weights of attributes from low to high. Except for the first layer model, the attributes with lower weights and the results of the upper layer are selected as input each time until the final state is reached. In this paper, a two-layer BRB model is constructed. Figure 4 is the structure of the hierarchical BRB model and the rule expression is profiled as:


(27) $$\begin{array}{c} R_{k}^{b}:\text{If}\;x_{1}\;\text{is}\;A_{1}^{k}\;\text{and}\;x_{2}\;\text{is}\;A_{2}^{k}\\ \text{Then}\;Y\;\text{is}\;\left\{\left(D_{1},\beta_{1,k}\right),\left(D_{2},\beta_{2,k}\right),\ldots ,\left(D_{N,}\beta_{N,k}\right)\right\}\\ \begin{array}{c} \text{With}\;k\;\text{rule weight}\;\theta_{k}\\ \text{and attribute weight}\;\delta_{1},\delta_{2}\; \end{array} \end{array}$$

where $R_{k}^{b}$ represents the kth rule of the bth layer model. Y represents the output result, which is also used as the input information of the next layer. $D_{j}$ represents the reference level of the output result, and $\beta_{j,k}$(j = 1, 2…, *N*) represents the belief degree of the jth level. $\theta_{k}$ represents the rule weight, $\delta_{1},\delta_{2}$ represents the attribute weight, and $\delta_{1}<\delta_{2}$.

###### 3.3.2.1.1. Construction of the first layer model BRB1

The importance of the three stock evaluation models is from high to low: ER_MA model, ER_MACD model, and ER_ KDJ model. Therefore, the MACD utility and KDJ utility are selected as the prerequisite attributes of the first-layer BRB model. The reference points are defined as follows:


(28) $$A_{1}\in \left\{N,M,P\right\}$$

(29) $$A_{2}\in \left\{N,M,P\right\}$$

$A_{1}$ is the MACD utility reference point, and $A_{2}$ is the KDJ utility reference point. The result $y_{1}\left(t\right)$ is the trading degree of the stock, and the reference point of $y_{1}\left(t\right)$ can be five reference points: buy all positions (BA), buy half positions (BH), hold a position (Hold), sell half positions(SH), and sell all positions (SA), which can be described as:


(30) $$\begin{array}{c} R_{k}^{1}:\text{If}\;z_{3}\left(t\right)\;\text{is}\;A_{1}^{k}\;\text{and}\;z_{2}\left(t\right)\;\text{is}\;A_{2}^{k}\\ \text{Then}\;y_{1}\left(t\right)\text{is}\;\left\{\left(SA,\beta_{1,k}\right),\left(SH,\beta_{2,k}\right),\left(\text{Hold},\beta_{3,k}\right),\left(BH,\beta_{4,k}\right),\left(BA,\beta_{5,k}\right)\right\}\\ \begin{array}{c} \text{with}\;k\;\text{rule weight}\;\theta_{k}\\ \text{and attribute weight}\;\delta_{1},\delta_{2} \end{array} \end{array}$$

where $R_{k}^{1}$ is the kth rule in model BRB1. $\beta_{i,k}$ represents the belief of the level corresponding to the rule. $\theta_{k}$ represents the weight of this rule. $\delta_{1}$ and $\delta_{2}$ represent attribute $z_{3}\left(t\right)$ and $z_{2}\left(t\right)$ weights.

###### 3.3.2.1.2. Construction of the second layer model BRB2

The result $y_{1}\left(t\right)$ obtained by the BRB1 model and the utility of the MA model are used as the prerequisite attributes to construct the second-layer model BRB2. The reference points are defined as follows:


(31) $$A_{3}\in \left\{N,M,P\right\}$$

(32) $$A_{4}\in \left\{SA,SH,\text{Hold},BH,BA\right\}$$

$A_{3}$ is the MA utility reference point, and $A_{4}$ is the $y_{1}\left(t\right)$ reference point. The result y(t) represents the final stock trading situation, and the reference point of y(t) can be five reference points: buy all positions (BA), buy half positions (BH), hold a position (Hold), sell half positions (SH), and sell all positions (SA). In the BRB2 model, there are 15 belief rules, which are described as:


(33) $$\begin{array}{c} R_{k}^{2}:\text{If}\;z_{1}\left(t\right)\;\text{is}\;A_{3}^{k}\;\text{and}\;y_{1}\left(t\right)\text{is}\;A_{4}^{k}\\ \text{Then}\;y\left(t\right)\;\text{is}\;\left\{\left(SA,\beta_{1,k}\right),\left(SH,\beta_{2,k}\right),\left(\text{Hold},\beta_{3,k}\right),\left(BH,\beta_{4,k}\right),\left(BA,\beta_{5,k}\right)\right\}\\ \begin{array}{c} \text{with}\;k\;\text{rule weight}\;\theta_{k}\\ \text{and attribute weight}\;\delta_{1},\delta_{2} \end{array} \end{array}$$

where $R_{k}^{2}$ is the kth rule in the BRB2 model. $\beta_{i,k}$ represents the belief of the level corresponding to the rule. $\theta_{k}$ represents the weight of the rule. $\delta_{1}$ and $\delta_{2}$ represent attribute weights.

##### 3.3.2.2. Reasoning process

**Figure 5:**
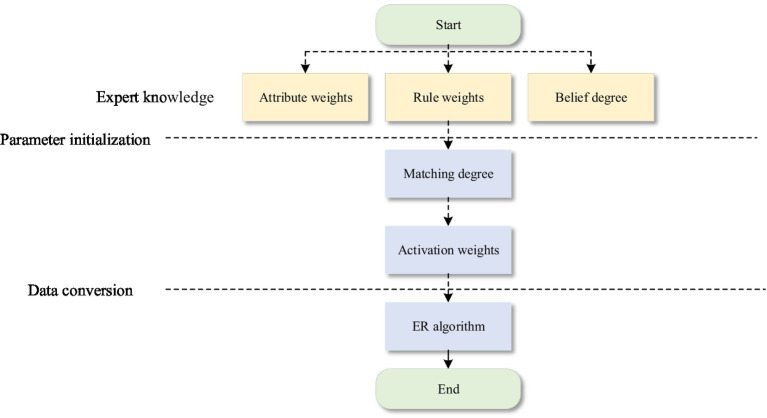
BRB model reasoning process.

First, the data are input into the designed belief rule base, and then the activated rules are combined by the ER analytic algorithm. Finally, the assessment results are output. The reasoning process is shown in Figure 5. The detailed process is as follows:

Step 1: In the BRB model, the input information needs to be transformed into a unified measurement framework, and the following formula is used to calculate the matching degree between the input and the reference value.


(34) $$\varphi_{j}^{i}=\{\begin{array}{ll} \frac{A_{i\left(k+1\right)}-z_{i}^{n}\left(t\right)}{A_{i\left(k+1\right)}-A_{i\left(k\right)}} & j=k,A_{i\left(k\right)}\leq z_{i}^{n}\left(t\right)\leq A_{i\left(k+1\right)}\\ \frac{z_{i}^{n}\left(t\right)-A_{i\left(k\right)}}{A_{i\left(k+1\right)}-A_{i\left(k\right)}} & j=k+1\\ 0 & j=1,2,\ldots L,j\neq k,k+1 \end{array}$$

where $\varphi_{j}^{i}$ denotes the matching degree of the jth attribute of the ith rule.

Step 2: After the matching degree is calculated, the calculated degree of activation of the input information to the rule is needed to calculate the rule activation weight. The formula is as follows:


(35) $$\tau_{k}=\frac{\theta_{k}\prod_{i=1}^{M}{\left(\varphi_{k}^{i}\right)}^{\delta_{i}}}{\sum_{l=1}^{L}\theta_{l}\prod_{i=1}^{M}{\left(\varphi_{k}^{l}\right)}^{\delta_{i}}},\;k=1,\ldots ,L$$

$\tau_{k}$ is the activation weight of the kth rule, $\theta_{k}$ is the rule weight of the kth rule, and $\delta_{i}$ is the attribute weight.

Step 3: After the evidence is activated and the activation weight of each rule is calculated, the belief distribution of the activated rule is combined using the ER analysis algorithm. The formulas are as follows:


(36) $$\beta_{n}=\frac{\mu \left[\prod_{k=1}^{L}\left(\tau_{k}\beta_{n,k}+1-\tau_{k}\sum_{j=1}^{N}\beta_{j,k}\right)-\prod_{k=1}^{L}\left(1-\tau_{k}\sum_{j=1}^{N}\beta_{j,k}\right)\right]}{1-\mu \left[\prod_{k=1}^{L}\left(1-\tau_{k}\right)\right]}$$

(37) $$\mu ={\left[\sum_{n=1}^{N}\prod_{k=1}^{L}\left(\tau_{k}\beta_{n,k}+1-\tau_{k}\sum_{j=1}^{N}\beta_{j,k}\right)-\left(N-1\right)\prod_{k=1}^{L}\left(1-\tau_{k}\sum_{j=1}^{N}\beta_{j,k}\right)\right]}^{-1}$$

Step 4: The final output of the stock forecast is $y\left(t\right)=\sum_{n=1}^{N}u\left(D_{n}\right)\beta_{n}$_._

y(t) is the actual output of the model, and $\beta_{n}$ is the belief of the estimated result $D_{n}$.

#### 3.3.3. Model optimization

Trading analysts make recommendations on trading behavior based on historical data, which can be verified by stock price movements and volume. These investment recommendations are used as actual values for the training of the model. The model constructed in this paper is an alternative to the process of investor analysis. To improve the accuracy of the model, it is necessary to train the model continuously. The goal of training is to find a set of parameters to minimize the difference between the predicted and actual values. The optimization model is described as follows:


(38) $$MSE\left(\eta \right)=\frac{1}{T}\sum_{t=1}^{T}{\left(y\left(t\right)-y_{\text{actual}}\left(t\right)\right)}^{2}$$

(39) $$\begin{array}{l} \;\;\min MSE\left(\eta \right)\\ 0\leq \theta_{k}\leq 1,\;\;k=1,2,\ldots L\\ 0\leq \beta_{n,k}\leq 1,\;n=1,\ldots ,N,\;\;\;k=1,2,\ldots L\\ \sum_{n=1}^{N}\beta_{n,k}\leq 1,\;\;\;k=1,2,\ldots ,L \end{array}$$

(40) $$\text{Accuracy}=\frac{\text{Acount}\left(aq\right)}{T}$$

where η is the set of parameters to be optimized. The set includes the belief degree, rule weight, and attribute weight. $y_{\text{actual}}\left(t\right)$ represents the actual investment recommendations of the stock market.y(t) denotes the investment recommendation output by the model. T represents the number of test data, and Acount represents the number of correct evaluation results.

In this paper, projection covariance matrix adaption evolution strategy (P-CMA-ES), which is an extension of CMA-ES algorithm is used for optimization. Compared with CMA-ES algorithm, P-CMA-ES algorithm reduces the complexity and improves the effectiveness of the optimization process (Yin et al., 2017). As an intelligent optimization algorithm for global optimization, P-CMA-ES can quickly converge to the global optimum without large sample set (Hu et al., 2020). It has significant advantages in small sample size and nonlinear optimization. The working process of the P-CMA-ES is shown in Figure 5. The optimization process is as follows:

Step 1: The dimension of the problem is defined, and the parameter set is initialized.

Step 2: Perform sampling operations and define the expected value. The covariance matrix of the corresponding population is generated by using the normal distribution. The formula is shown below.


(41) $$\eta_{i}^{g+1}\sim {\text{mean}}^{g}+{℘}^{g}N\left(0,C^{g}\right)\left(i=1\cdots ƛ\right)$$

$\eta_{i}^{g+1}$ represents the ith descendant of the g+1th generation. ${\text{mean}}^{g}$ represents the expected value of the g-th generation. ${℘}^{g}$ is the step size of the gth generation. $N\left(0,C^{g}\right)$ is the orthogonal distribution, and $C^{g}$ represents the covariance matrix of the population.

Step 3: Based on the projection algorithm, the solution is mapped to the hyperplane, and the parameters are constrained. The formula is shown below.


(42) $$\begin{array}{l} \eta_{i}^{g+1}\left(1+V_{n}\times \left(j-1\right):V_{n}\times j\right)\\ =\eta_{i}^{g+1}\left(1+V_{n}\times \left(j-1\right):V_{n}\times j\right)-P_{n}^{T}\times {\left(P_{n}\times P_{n}^{T}\right)}^{-1}\\ \times \eta_{i}^{g+1}\left(1+V_{n}\times \left(j-1\right):V_{n}\times j\right)\times P_{n} \end{array}$$

$P={\left[1\cdots 1\right]}_{1\times N}$ represents the parameter vector. $V_{n}=\left(1,\cdots ,R\right)$ is the number of constraint variables. j=(1,⋯,R+1) is the number of constraints.

Step 4: The subpopulations that meet the constraints are selected. The formula is described as follows.


(43) $${\text{mean}}^{g+1}=\sum_{i=1}^{\alpha }\varpi_{i}\eta_{i}^{g+1}$$

$\varpi_{i}$ denotes the weight of the optimal solution, and all weights add up to 1.

Step 5: The covariance matrix of the subpopulation is updated and gradually approaches the optimal solution, which process is described as follows:


(44) $$\begin{array}{l} C^{g+1}=\left(1-b_{1}-b_{\alpha }\right)C^{g}+b_{1}J^{g+1}{\left(J^{g+1}\right)}^{T}\\ +b_{\alpha }\sum_{i=1}^{\alpha }\varpi_{i}\left(\frac{\eta_{i}^{g+1}-{\text{mean}}^{g}}{{℘}^{g}}\right){\left(\frac{\eta_{i}^{g+1}-{\text{mean}}^{g}}{{℘}^{g}}\right)}^{T} \end{array}$$

(45) $$J_{s}^{g+1}=\left(1-b_{s}\right)J_{s}^{g}+\sqrt{b_{s}\left(2-b_{s}\right){\left(\sum_{i=1}^{\alpha }\varpi_{i}^{2}\right)}^{-1}}\times \frac{{\text{mean}}^{g+1}-{\text{mean}}^{g}}{{℘}^{g}}$$

(46) $${℘}^{g+1}={℘}^{g}\exp\left(\frac{b_{℘}}{d_{℘}}\left(\frac{\left||J_{℘}^{g+1}|\right|}{E||N\left(0,Ι\right)||}-1\right)\right)$$

where $b_{1}$ and $b_{\alpha }$ represents the learning rate, $J^{g}$ is the evolutionary path of the gth generation, and $d_{℘}$ is the damping coefficient. E||N(0,Ι)|| represents the expected value of a normal distribution N(0,Ι), where Ι is the identity matrix.

Remark 3: The hierarchical BRB is easy to extend. When the model needs to add new indicators, it can be achieved by increasing the level of the model. Each layer of the model contains two indicators, which facilitates the analysis of the relationship between the indicators. The analysis process is more organized.

Remark 4: The BRB uses the ER algorithm as the inference engine, and expert knowledge is introduced. Therefore, the validity of the decision-making results is guaranteed. In addition, a BRB is established based on IF-THEN rules to make the reasoning process interpretable.

## 4. Case study

The Shanghai Composite Index is a comprehensive index of all stocks, which can reflect the situation of the entire securities market, thereby providing investors with different investment references. Therefore, in this section, the Shanghai Composite Index is used as the research object to analyze stock market sentiment. The stock data are a time series, and a continuous data set of stock market opening days in 2010–2019 is used in this paper. The data contain four variables: opening price, closing price, high price and low price. Although there are many data in this range, there are few data with decision-making significance and buying and selling signals. Additionally, when calculating indicators such as MA and MACD and actually analyzing stocks, investors need to refer to multiple days of data to arrive at an investment strategy. Therefore, this experiment analyzes 390 days of stock market trading with a time interval of 5–9 days.

The stock market evaluation model is introduced in Section 4.1. Based on the results of different types of assessments, comprehensive decisions about the stock market are conducted in Section 4.2. The experimental results are analyzed in Section 4.3, and the effectiveness of the model is demonstrated. The experimental results are analyzed in Section 4.3. The effectiveness of the model is proved.

### 4.1. The stock market evaluation model

First, we build a primary stock market model. According to the classification of technical indicators, models are established to fuse data. Then, based on this model, the utility obtained quantifies the state of the stock market. In the actual analysis of stocks, it is impossible to judge trading behavior only by relying on quantitative data. Therefore, the relationship between the data are compared at this time. In addition, the stock data are a time series, and the golden cross needs to be judged with reference to the previous day’s data. Therefore, it is necessary to convert part of the quantitative data into qualitative data as input to the model. The influence of technical indicators on the state of the stock market can be divided into three levels, namely, positive signal, intermediate signal and negative signal. Based on Section 3 analysis, 5-day deviation, MACD deviation and K-D were quantitative data, combined with expert knowledge to set reference values such as Table 4.

**Table 4:** Reference value of quantitative data.

Attribute	Reference 1	Reference 2	Reference 3
5MA deviation	-2	0	2
MACD deviation	-5	0	5
K-D	−13	0	13

### 4.2. Stock market comprehensive decision-making model

The stock market comprehensive decision-making model is defined in Section 3.2 of this paper. In BRB1, premise attributes $z_{3}\left(t\right)$ and $z_{2}\left(t\right)$ are the output utility of the ER_MACD and ER_KDJ models, respectively, and the reference points of premise attributes are shown in Table 5 and Table 6. The reference point of the output $y_{1}\left(t\right)$ of the BRB1 model is displayed in Table 7. In BRB2, the BRB1 model output $y_{1}\left(t\right)$ is the premise attribute input, and the attribute $z_{1}\left(t\right)$ is the ER_MA model output utility. The model outputs y(t), and the reference value is displayed in Table 8, 9. The initial belief rules for BRB1 and BRB2 are shown in Tables 10, 11.

**Table 5:** The reference point of $z_{3}\left(t\right)$_._

Reference point	*P*	*M*	*N*
Reference value	1	3	5

**Table 6:** The reference points of $z_{2}\left(t\right)$_._

Reference point	*P*	*M*	*N*
Reference value	1	3	5

**Table 7:** The reference point of $y_{1}\left(t\right)$_._

Reference point	BA	BH	Hold	SH	SA
Reference value	1	2	3	4	5

**Table 8:** The reference point of $z_{1}\left(t\right)$_._

Reference point	*P*	*M*	*N*
Reference value	1	3	5

**Table 9:** The reference point of y(t)_._

Reference point	BA	BH	Hold	SH	SA
Reference value	1	2	3	4	5

**Table 10:** BRB1 initial belief rule base.

No.	Attribute	Rule weight	Output belief degree $\left\{D_{1},D_{2},D_{3},D_{4},D_{5}\right\}$	
1	N	N	1	{1,0,0,0,0}
2	N	M	1	{0.3,0.6.0.1,0,0}
3	N	P	1	{0.15,0.3,0.4,0.1,0.05}
4	M	N	1	{0.1,0.3,0.6,0,0}
5	M	M	1	{0,0,1,0,0}
6	M	P	1	{0,0,0.6,0.3,0.1}
7	P	N	1	{0.05,0.1,0.4,0.3,0.15}
8	P	M	1	{0,0,0.3,0.5,0.2}
9	P	P	1	{0,0,0,0,1}

**Table 11:** BRB2 initial belief rule base.

No.	Attribute	Rule weight	Output belief degree $\left\{D_{1},D_{2},D_{3},D_{4},D_{5}\right\}$	
1	N	SA	1	{1,0,0,0,0}
2	N	SH	1	{0.6,0.4,0,0,0}
3	N	Hold	1	{0.3,0.5,0.2,0.0,0}
4	N	BH	1	{0.1,0.3,0.6,0,0}
5	N	BA	1	{0,0.2,0.7,0,0}
6	M	SA	1	{0,0.2,0.7,0,0}
7	M	SH	1	{0.1,0.3,0.6,0,0}
8	M	Hold	1	{0,0,1,0,0}
9	M	BH	1	{0,0,0.6,0.2,0.1}
10	M	BA	1	{0,0,0.7,0.2,0.1}
11	P	SA	1	{0,0.1,0.6,0.2,0.1}
12	P	SH	1	{0,0.05,0.6,0.25,0.1}
13	P	Hold	1	{0,0,0.2,0.5,0.3}
14	P	BH	1	{0,0,0,0.4,0.6}
15	P	BA	1	{0,0,0,0,1}

Finally, based on the above model, five kinds of labels can be output to represent the investment recommendations. The meaning of the labels is displayed in Table 12, and the definition formula for the five labels is shown as:


(47) $$\text{label}=\{\begin{array}{l} 1,y\leq 1.5\\ 2,1.5\leq y\leq 2.5\\ 3,2.5\leq y\leq 3.5\\ 4,3.5\leq y\leq 4.5\\ 5,4.5\leq y \end{array}$$

**Table 12:** The meaning of labels.

Label	1	2	3	4	5
Meaning	Buy all positions	Buy half positions	Hold	Sell half positions	Sell all positions

### 4.3. Simulation experiment

**Figure 6:**
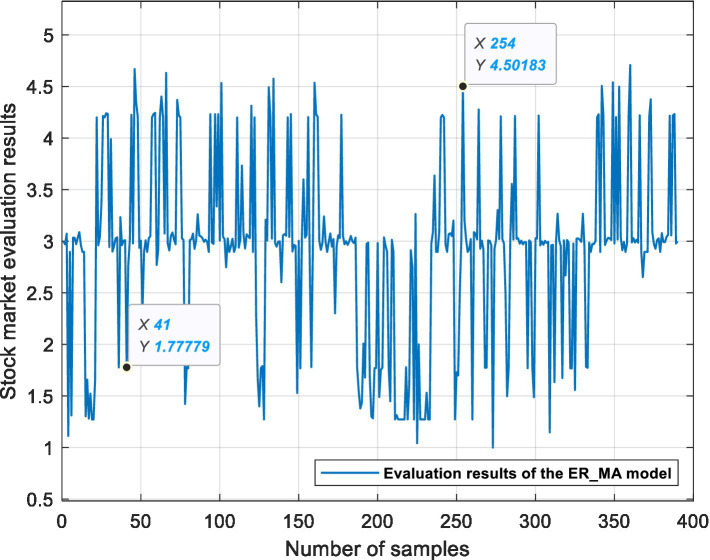
ER_MA model stock market evaluation results.

**Figure 7:**
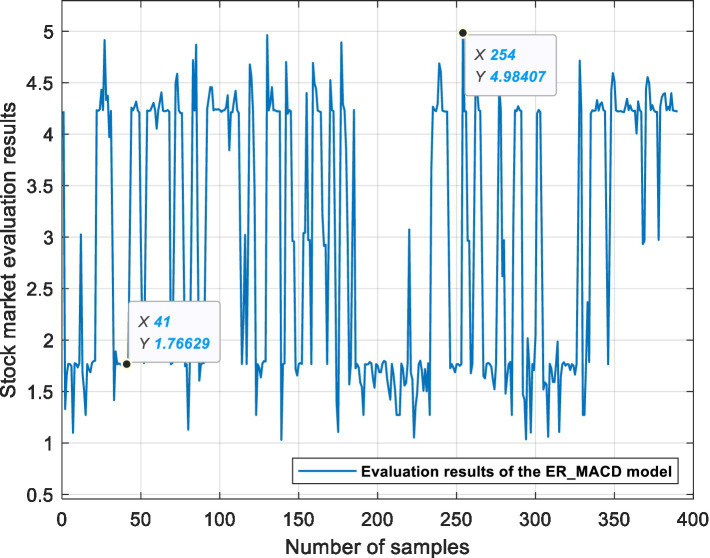
ER_MACD model stock market evaluation results.

**Figure 8:**
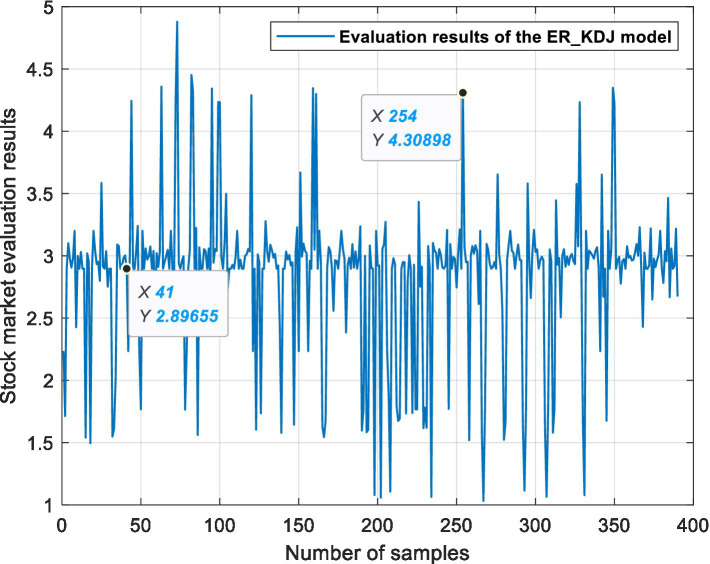
ER_KDJ model stock market evaluation results.

This experiment is based on three ER stock market evaluation models, which are used for data fusion, processing semi-quantitative information and quantifying the state of the stock market. Figures 6, 7, 8 show the evaluation results of the ER_MA, ER_MACD, and ER_KDJ models, respectively. In this section, two sample data are selected as examples to analyze the evaluation process of the stock market.

Example 1: Take the 41st sample data, that is, the Shanghai Stock Exchange Index on April 20, 2011, as an example. After calculation, the data of this day are shown in Table 13. The data show 10 MA >20 MA > 60 MA, which is rated as a positive signal. There is no crossing between 5 MA and 10 MA, and the 5MA_ deviation is divided into intermediate signals. Therefore, according to the influence of these three attributes on the stock market, the ER_MA model evaluates that the current market is in a positive state. Investors can consider buying half a position. The ER_MACD model is analyzed in the same way. In the ER_KDJ model, the K-D value attribute is divided into negative signals, and there is no crossing between the K value and D value. Therefore, the model evaluates that the current market is in an intermediate state, and investors can consider taking a position and waiting.

**Table 13:** Data from 2011/4/20.

Indicator	Value
Close price	3007.649
5MA	3036.645
10MA	3179.322
20MA	3210.665
60MA	3473.137
5MA_ deviation	−0.955
DIF	−139.8
DEA	−83.9026
MACD	−111.795
K	11.967
D	13.527
K-D	−1.558

Example 2: Take the 254th sample data, that is, the Shanghai Stock Exchange Index on January 14, 2016, as an example. After calculation, the data of this day are shown in Table 14. The data show that 10 MA < 20 MA <60 MA. There is no crossing between 5 MA and 10 MA, and the 5 MA_ deviation is divided into intermediate signals. At this time, based on the ER_MA model, the current market is in a negative state, and investors need to consider reducing their positions. For the ER_MACD model, the data show that DIF < 0, and DEA < 0. DIF and DEA have formed a dead cross, and the deviation degree of MACD shows a negative signal. Therefore, this model evaluates that the current stock market is in a negative state, and investors can consider selling stocks. By the same token, the ER_KDJ model can evaluate that the current market is in a negative state at this time.

**Table 14:** Data from 2016/1/14.

Indicator	Value
Close price	3007.037
5MA	3031.314
10MA	3028.901
20MA	2993.794
60MA	2903.931
5MA_ deviation	−0.80086
DIF	28.895
DEA	30.548
MACD	−3.306
*K*	48.214
*D*	67.991
K-D	−19.778

**Figure 9:**
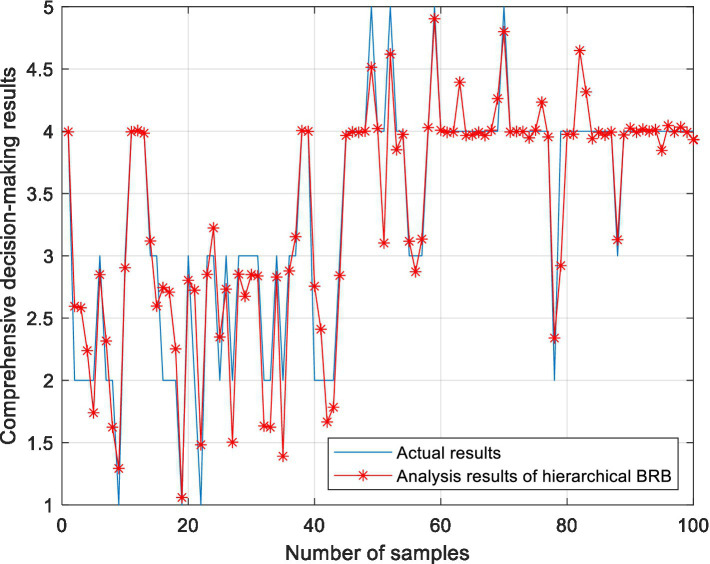
Analysis results of the hierarchical BRB model.

**Figure 10:**
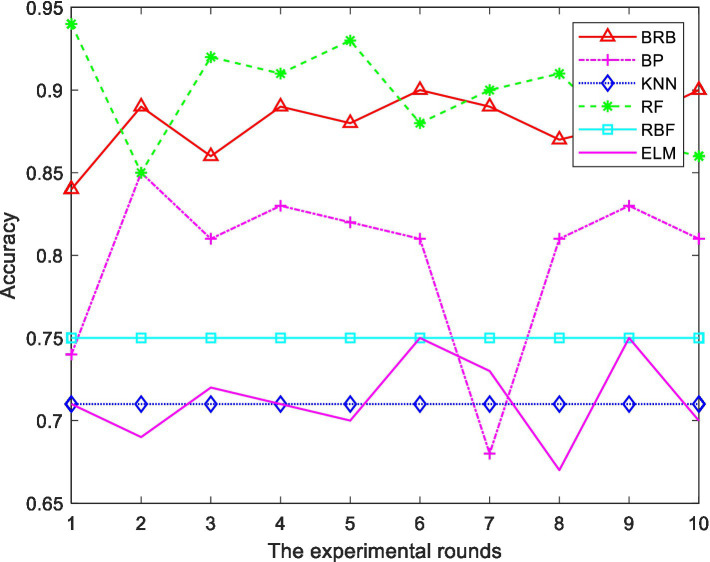
Comparison of different methods.

In the BRB1 model, the full set is used for testing. In the BRB2 model, 290 pieces of data are trained, and 100 pieces of data are test sets. To prove the effectiveness of the model, 10 rounds of experiments were carried out. For the hierarchical BRB model, the maximum number of iterations was 300. Figure 9 shows the results of the comprehensive analysis of stocks. The average value of 10 rounds of experiments represents the accuracy of the model. To further verify the effectiveness of the methods used in this paper, this model is compared with the BP neural network, ELM, RF and RBF. To facilitate the comparison of the experiment, 200 pieces of data are also selected as the training set, and 190 pieces of data are selected as the test set. Ten rounds of experiments were carried out, and the accuracy of the model was the average of 10 rounds of experiments. The accuracy of 10 rounds of experiments with 5 methods is shown in Figure 10. The accuracy of each method is shown in Table 15. The optimized belief rule base is shown in Tables 16, 17.

**Table 15:** Accuracy of different methods.

Method	BRB	BP	ELM	RF	RBF
Accuracy	0.88	0.799	0.713	0.897	0.75

**Table 16:** BRB1 optimized belief rule base.

No.	Attribute	Rule weight	Output belief degree $\left\{D_{1},D_{2},D_{3},D_{4},D_{5}\right\}$	
1	N	N	0.4144	{0.7071,0.2855,0.0075,0.0059,0}
2	N	M	0.7060	{0.7379,0.15066,0.0830,0.0039,0.0243}
3	N	P	0.9138	{0.0094,0.0146,0.0304,0.4565,0.4888}
4	M	N	0.4091	{0.0320,0.2179,0.2862,0.3159,0.1477}
5	M	M	0.7859	{0.2445,0.0067,0.3502,0.3685,0.0299}
6	M	P	0.1194	{0.1952,0.2250,0.0214,0.3974,0.1607}
7	P	N	0.1803	{0.1350,0.0217,0.3531,0.2848,0.2051
8	P	M	0.9467	{0.0250,0.1073,0.0594,0.0754,0.7326}
9	P	P	0.0082	{0.1933,0.0100,0.0026,0.1833,0.6105}

**Table 17:** BRB2 optimized belief rule base.

No.	Attribute	Rule weight	Output belief degree $\left\{D_{1},D_{2},D_{3},D_{4},D_{5}\right\}$	
1	N	SA	0.9090	{0.9832,0.0026,0.0100,0.0045,0}
2	N	SH	0.1470	{0.3308,0.2002,0.0579,0.3232,0.0879}
3	N	Hold	0.2917	{0.3007,0.1356,0.3115,0.1057,0.1463}
4	N	BH	0.8390	{0.9575,0.03057,0,0,0.01801}
5	N	BA	0.1592	{0.0323,0.2352,0.2309,0.3203,0.1810}
6	M	SA	0.2793	{0.6336,0,0.0090,0.16237,0.19607}
7	M	SH	0.3369	{0.4571,0.2371,0.1397,0.1615,0.0044}
8	M	Hold	0.2238	{0.14863,0.0335,0.65614,0.10714,0.054533}
9	M	BH	0.4560	{0.0969,0.0024,0.6625,0.1246,0.1134}
10	M	BA	0.7037	{0.0016,0.0109,0.0042,0.6156,0.3674}
11	P	SA	0.8437	{0.6596,0.0100,0.1618,0.15869,0.0097}
12	P	SH	0.4753	{0.2222,0.039375,0.2815,0.2148,0.24188}
13	P	Hold	0.9859	{0.04514,0.1047,0.0911,0.1078,0.6511}
14	P	BH	0.4233	{0.0001,0.0011,0.0022,0.0028,0.9936}
15	P	BA	0.8160	{0.2910,0.0499,0.1105,0.2822,0.2662}

The above experiments show that, compared with other methods, the model constructed in this paper has certain advantages. The description is as follows:

1. In 10 rounds of experiments, the accuracy of the ER and hierarchical BRB model constructed in this paper is between 88 and 91%, indicating the feasibility of the model.
2. As can be seen from Figure 10, RF also performs well in terms of prediction accuracy. However, RF is regarded as a black box, and the intermediate process of decision making is difficult to explain. Although the accuracy is high, it does not explain the reason for the decision. Moreover, when the number of decision trees in a random forest is large, the space and time required for training is relatively large. However, the reasoning process of BRB is transparent and the model can be interpreted. The process of model construction is based on expert knowledge. The results are more interpretable and reliable. Therefore, it is more suitable for analyzing the field of stock investment with risks.
3. Compared to other methods, this model can comprehensively handle quantitative and qualitative information.

## 5. Conclusion

This paper proposed a stock analysis model based on ER and hierarchical BRB, which can not only analyze the stock market from both quantitative and qualitative perspectives, but also solve the problem of rule explosion caused by multiple attribute inputs in traditional BRB models. In addition, the whole reasoning process of the model is relatively transparent, and the decision results can be interpreted. The experimental results show that the proposed model is suitable for analyzing this kind of investment decision making problems under uncertain and risky environment. In the future, the following areas of work need to be enhanced:

1. In the in-depth study of the stock market, the impact of uncertain factors is considered, such as national policies, market supply and demand. In the future, a standard stock market buying and selling decision-making system needs to be constructed.
2. The optimization model in this paper is based on the P-CMA-ES algorithm, which is a global optimization algorithm. The optimization process does not consider the meaning of the optimization parameters, and the optimization process lacks interpretability. Therefore, ensuring the interpretability of the optimization process is a problem that needs to be solved.

## Data availability statement

The original contributions presented in the study are included in the article/supplementary material, further inquiries can be directed to the corresponding authors.

## Author contributions

YC and JL participated in the conceptualization. YG, WH and HL were in charge of the methodology and software. GZ and HW performed validation, formal analysis. First draft writing, review, and editing were handled by YC, JL, and WH. All authors contributed to the article and approved the submitted version.

## Funding

This work was supported in part by the Natural Science Foundation of China under Grant 62203461 and Grant 62203365, in part by the Postdoctoral Science Foundation of China under Grant No. 2020 M683736, in part by the Teaching reform project of higher education in Heilongjiang Province under Grant Nos. SJGY20210456 and SJGY20210457, in part by the Natural Science Foundation of Heilongjiang Province of China under Grant No. LH2021F038, in part by the graduate academic innovation project of Harbin Normal University under Grant No. HSDSSCX2022-18, in part by the Foreign Expert Projects in Heilongjiang under Grant No. GZ20220131.

## Conflict of interest

The authors declare that the research was conducted in the absence of any commercial or financial relationships that could be construed as a potential conflict of interest.

## Publisher’s note

All claims expressed in this article are solely those of the authors and do not necessarily represent those of their affiliated organizations, or those of the publisher, the editors and the reviewers. Any product that may be evaluated in this article, or claim that may be made by its manufacturer, is not guaranteed or endorsed by the publisher.
